# Pulmonary Rehabilitation Using Minimal Equipment for People With Chronic Obstructive Pulmonary Disease: A Systematic Review and Meta-Analysis

**DOI:** 10.1093/ptj/pzad013

**Published:** 2023-02-09

**Authors:** Sonia Wing Mei Cheng, Zoe J McKeough, Renae J McNamara, Jennifer A Alison

**Affiliations:** Discipline of Physiotherapy, Sydney School of Health Sciences, Faculty of Medicine and Health, The University of Sydney, Sydney, Australia; Discipline of Physiotherapy, Sydney School of Health Sciences, Faculty of Medicine and Health, The University of Sydney, Sydney, Australia; Department of Physiotherapy, Prince of Wales Hospital, South Eastern Sydney Local Health District, Sydney, Australia; Woolcock Emphysema Centre, Woolcock Institute of Medical Research, Sydney, Australia; Discipline of Physiotherapy, Sydney School of Health Sciences, Faculty of Medicine and Health, The University of Sydney, Sydney, Australia; Allied Health Professorial Unit, Sydney Local Health District, Sydney, Australia

**Keywords:** Chronic Obstructive Pulmonary Disease, Equipment and Supplies, Exercise, Pulmonary Rehabilitation

## Abstract

**Objective:**

Pulmonary rehabilitation programs that use minimal equipment for exercise training, rather than gymnasium equipment, would enable delivery of pulmonary rehabilitation to a greater number of people with chronic obstructive pulmonary disease (COPD). The effectiveness of minimal equipment programs in people with COPD is unclear. This systematic review and meta-analysis aimed to determine the effects of pulmonary rehabilitation using minimal equipment for aerobic and/or resistance training in people with COPD.

**Methods:**

Literature databases were searched up to September 2022 for randomized controlled trials (RCTs) comparing the effect of minimal equipment programs with usual care or with exercise equipment-based programs for exercise capacity, health-related quality of life (HRQoL), and strength.

**Results:**

Nineteen RCTs were included in the review and 14 RCTs were included in the meta-analyses, which reported low to moderate certainty of evidence. Compared with usual care, minimal equipment programs increased 6-minute walk distance (6MWD) by 85 m (95% CI = 37 to 132 m). No difference in 6MWD was observed between minimal equipment and exercise equipment-based programs (14 m, 95% CI = −27 to 56 m). Minimal equipment programs were more effective than usual care for improving HRQoL (standardized mean difference = 0.99, 95% CI = 0.31 to 1.67) and were not different from exercise equipment-based programs for improving upper limb strength (6 N, 95% CI = −2 to 13 N) or lower limb strength (20 N, 95% CI = −30 to 71 N).

**Conclusion:**

In people with COPD, pulmonary rehabilitation programs using minimal equipment elicit clinically significant improvements in 6MWD and HRQoL and are comparable with exercise equipment–based programs for improving 6MWD and strength.

**Impact:**

Pulmonary rehabilitation programs using minimal equipment may be a suitable alternative in settings where access to gymnasium equipment is limited. Delivery of pulmonary rehabilitation programs using minimal equipment may improve access to pulmonary rehabilitation worldwide, particularly in rural and remote areas and in developing countries.

## Introduction

Pulmonary rehabilitation is a highly effective therapeutic strategy for improving exercise capacity, health-related quality of life (HRQoL), and strength and dyspnea and for reducing hospitalizations and hospital readmissions following an exacerbations in people with chronic obstructive pulmonary disease (COPD).^1–3^ The key component of pulmonary rehabilitation is individually tailored, progressive aerobic and resistance exercise training. Clinical practice guidelines from major respiratory organizations recommend that aerobic training be performed at more than 60% of peak work rate from an incremental exercise test for at least 20–30 minutes and at a frequency of 3–5 times per week to increase endurance capacity and reduce dyspnea.^4–7^ Resistance training of the large muscle groups should be performed at an intensity of 8 to 10 repetition maximum (RM) 2 to 3 times per week, progressing from 1 to 3 sets, to optimize peripheral muscle strength.^4–7^ Most pulmonary rehabilitation programs take place in hospital gymnasiums and may use treadmills, stationary cycles, and arm and rowing ergometers for aerobic training, and weight machines for resistance training.^8^

Globally, the demand for pulmonary rehabilitation far exceeds the availability of programs. Only 2% to 5% of people with symptomatic COPD who would benefit from pulmonary rehabilitation are estimated to have access to programs.^9–12^ In both low-middle-income and high-income countries, lack of access to pulmonary rehabilitation is directly attributed to inadequate local infrastructure and funding, where it is not always feasible to deliver pulmonary rehabilitation programs in gymnasiums that are well resourced with exercise equipment.^13–15^ Randomized controlled trials (RCTs) with small samples of people with COPD have evaluated the effectiveness of exercise training programs using minimal exercise equipment, including ground walking^16^ and Nordic walking^17^ for aerobic training, and hand weights, body weight resistance exercises,^18^ and elastic resistance band exercises^19^ for resistance training. These individual studies provide preliminary evidence that compared with no exercise training, minimal equipment training programs can elicit clinically meaningful improvements in exercise capacity, muscle strength, HRQoL, and dyspnea in people with COPD. A propensity-matched analysis of 318 people with COPD undergoing supervised pulmonary rehabilitation using minimal equipment reported similar clinically significant improvements in exercise capacity and HRQoL that were non-inferior to pulmonary rehabilitation delivered using specialist exercise equipment.^20^ If pulmonary rehabilitation programs using minimal equipment for aerobic and resistance training are proven to be effective, this would support the delivery of pulmonary rehabilitation at centers with limited access to exercise equipment and in home-based and telerehabilitation-based settings. Widespread delivery of minimal equipment programs may increase the availability of pulmonary rehabilitation to a greater number of people with COPD, particularly those living in rural and remote areas or in developing countries.

To our knowledge, no review has rigorously evaluated whether pulmonary rehabilitation programs using minimal equipment can achieve similar benefits to conventional programs using exercise equipment in people with COPD. Previous reviews conducted more than 5 years ago compared minimal equipment programs with usual care only and included training programs of insufficient dosage and/or supervision to elicit skeletal muscle adaptations in people with COPD.^21^ The purpose of this systematic review was to pool results from RCTs on the effects of pulmonary rehabilitation using minimal equipment for aerobic and/or resistance training in people with COPD to determine whether minimal equipment programs lead to clinically relevant improvements in exercise capacity, HRQoL, muscle strength, dyspnea, anxiety, and depression and whether they are comparable with exercise equipment–based pulmonary rehabilitation programs.

## Methods

### Data Sources and Searches

A systematic literature search was initially undertaken in July 2021 and later updated in September 2022. The search was conducted using Cochrane Central Register of Controlled Trials, Medline, EMBASE, PsycINFO, Cumulative Index to Nursing and Allied Health Literature, and Allied and Complementary Medicine databases from inception to present. The detailed search terms are listed in the Supplementary Appendix 1. The reference lists of all included studies and review articles were hand searched for additional references.

**Figure 1:**
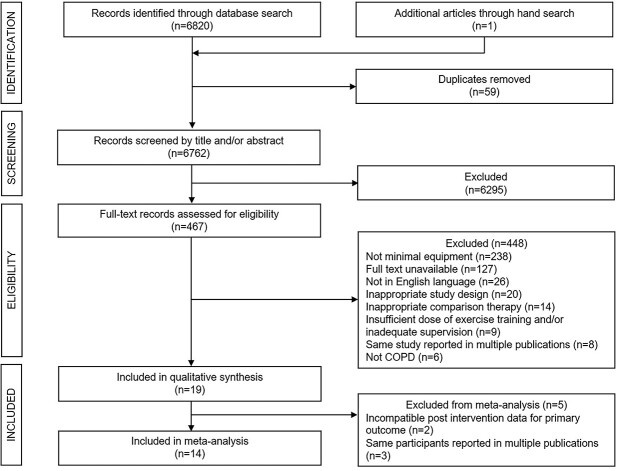
PRISMA flow diagram of screening, selection process, and inclusion of studies.

Criteria for study inclusion were English language full-text publications that (1) involved adults with a clinical diagnosis of COPD who were stable or recently had an exacerbation; (2) used an RCT design to evaluate the effectiveness of a pulmonary rehabilitation program using minimal equipment for aerobic training, resistance training, or both, of at least 4 weeks in duration with supervised exercise sessions provided at least twice per week; and (3) compared minimal equipment exercise training with usual care or with exercise equipment–based training. Minimal equipment programs were defined as exercise training programs that did not use any of the following: treadmills, leg or arm cycle ergometers, rowing machines, weight machines, or videoconferencing technology in home-based pulmonary rehabilitation or telerehabilitation settings. Parallel-group studies, but not crossover studies, were included. Studies that provided other components of pulmonary rehabilitation (eg, education, airway clearance, inspiratory muscle training) were included if the components were not part of the randomized treatment. Reporting outcomes of interest was not an inclusion criterion. Studies that did not provide exercise training at an intensity known to elicit skeletal muscle adaptations (ie, <50% of peak exercise capacity, symptoms below a “moderate” level on the Borg 0–10 category ratio scale,^22^ no indication of training intensity) were excluded. Studies evaluating water-based exercise training and tai χ were excluded because systematic reviews on the effectiveness of these specific training modalities have already been conducted in people with COPD.^23^^,^^24^

This systematic review was prospectively registered at PROSPERO (www.crd.york.ac.uk/PROSPERO) as CRD42021048159.

### Study Selection

The review authors (S.C., Z.M., R.M., and J.A.) independently screened all identified publications for inclusion based on the titles, abstracts, or full texts. Each publication was screened by 2 authors, with disagreements resolved through discussion or by consultation with a third author.

### Data Extraction and Quality Assessment

The authors independently extracted the following data from the included studies: first author; publication year; sample size; participant demographics, inclusion of stable COPD or recent exacerbation of COPD; modality, intensity, frequency, duration, progression, and length of minimal equipment training program; comparison therapy; primary and secondary outcome measures; and results. A second author spot-checked accuracy of extracted data against the original publication.

Two authors independently assessed risk of bias for each study using the Cochrane Risk of Bias tool.^25^ Two authors independently rated the certainty of evidence using the Grading of Recommendations Assessment, Development, and Evaluation approach.^26^ Certainty of evidence was graded as high, moderate, low, or very low and was downgraded if there were concerns with risk of bias, inconsistency, imprecision, and indirectness. Disagreements were resolved by discussion or by consultation with a third author. Because no meta-analysis had 10 or more studies, publication bias could not be reliably assessed.

### Data Synthesis and Analysis

The primary outcome in the meta-analysis was 6-minute walk distance (6MWD) at the completion of exercise training (ie, the difference in 6MWD immediately postintervention between the minimal equipment group and comparison group), which is recommended by the Cochrane Collaboration to reduce measurement error.^25^ Secondary outcomes were postintervention incremental shuttle walk test (ISWT) distance, endurance shuttle walk test (ESWT) time, lower limb and upper limb muscle strength, HRQoL, dyspnea, anxiety, and depression. Effect sizes were pooled across studies that compared minimal equipment programs with usual care separately with studies that compared minimal equipment programs with exercise equipment–based programs. A random effects model was chosen due to differences in dosage and length of exercise training interventions across the included studies. Subgroup analyses were performed based on type of training (aerobic, resistance, or combined). For studies that reported change scores in addition to postintervention scores, sensitivity analyses comparing minimal equipment programs with usual care and with equipment-based programs were performed using the same methods as the main analyses. Heterogeneity among the studies in each analysis was assessed using the I^2^ statistic. *P* <.05 was considered statistically significant. All statistical analyses were performed using Review Manager (RevMan) version 5.4.^25^

## Results

### Search Results and Eligible Studies

The database searches identified 6820 publications and 1 publication was identified through hand search, of which 6295 records were excluded based on title and/or abstract, leaving 467 full-text records; these were assessed for eligibility. Nineteen studies met the inclusion criteria.^16–19^^,^^27–41^ Fourteen RCTs involving 580 participants could be included in the quantitative meta-analysis.^16–19^^,^^29–35^^,^^37^^,^^38^^,^^41^ Characteristics of the included studies are summarized in the Supplementary Table.

The included studies were published between 1996 and 2020 and were conducted in Australia, Brazil, China, Europe, New Zealand, Turkey, and the United States. Sample sizes of each RCT ranged from 19 to 130 participants, the majority of whom had stable COPD. The mean ± SD age of participants was 67 ± 3 years and mean forced expiratory volume in 1 second (FEV_1_) was 49 ± 7% predicted. Approximately 65% of participants were male. Of the 19 RCTs, 18 were conducted in hospital- or university-based outpatient settings and 1 in the community setting. Nine studies evaluated the effects of minimal equipment programs compared with usual care, of which 3 studies conducted aerobic training only,^16^^,^^17^^,^^29^ 1 conducted resistance training only,^34^ and 5 conducted combined aerobic and resistance training.^27^^,^^28^^,^^31^^,^^33^^,^^41^ Ten studies compared minimal equipment programs with exercise equipment–based programs, of which 1 study conducted aerobic training,^32^ 8 conducted resistance training,^19^^,^^30^^,^^35–40^ and 1 conducted combined training.^18^ Minimal equipment training modalities included ground walking,^16^^,^^28^^,^^29^^,^^31^^,^^32^ Nordic walking,^17^^,^^18^ and stair climbing^27^^,^^33^ for aerobic training, and elastic resistance bands,^30^^,^^34^^,^^39–41^ elastic resistance tubes,^19^^,^^27^^,^^30^^,^^35–40^ hand-held weights,^28^ and body weight exercises^18^^,^^33^^,^^41^ for resistance training. One RCT evaluated 2 different types of minimal equipment resistance training (ie, elastic bands and elastic tubes) compared with exercise equipment–based training, and both minimal equipment groups were included in the meta-analyses.^30^

All studies provided exercise training at an intensity known to produce training effects in people with COPD. Aerobic training in the included studies was prescribed using 75% to 80% of initial 6-Minute Walk Test or ISWT speed or using a score of 3 to 4 on the 0 to 10 modified Borg dyspnea scale. Resistance training in the included studies was most commonly prescribed at an initial intensity of 10 to 15 RM with elastic resistance bands or tubing. The duration of exercise sessions varied from 20 minutes in 2 studies,^27^^,^^28^ which were not included in the quantitative meta-analysis, up to 60 minutes. One study did not report duration of the exercise sessions.^17^

Common progressions for aerobic training included increased duration of the session,^28^^,^^29^ increased walking speed,^16^^,^^32^ and addition of weights when walking^16^^,^^32^ to maintain adequate intensity measured by heart rate, perceived dyspnea, or rate of perceived exertion. Common progressions for resistance training included: increased repetitions and sets^19^^,^^30^^,^^34^^,^^35^^,^^39^^,^^40^; heavier weights^28^; higher resistance elastic bands/tubing by increasing their thickness, diameter, and/or adding additional bands/tubing^30^^,^^37^^,^^38^; and increased intensity measured by RM testing.^37^^,^^38^ One study conducting aerobic training^17^ and 5 studies conducting combined aerobic and resistance training^18^^,^^27^^,^^31^^,^^33^^,^^41^ did not report the method of exercise progression. Length of the minimal equipment programs was commonly 8 or 12 weeks, with a range of 6 weeks to 28 weeks.

### Quality of Studies

**Figure 2:**
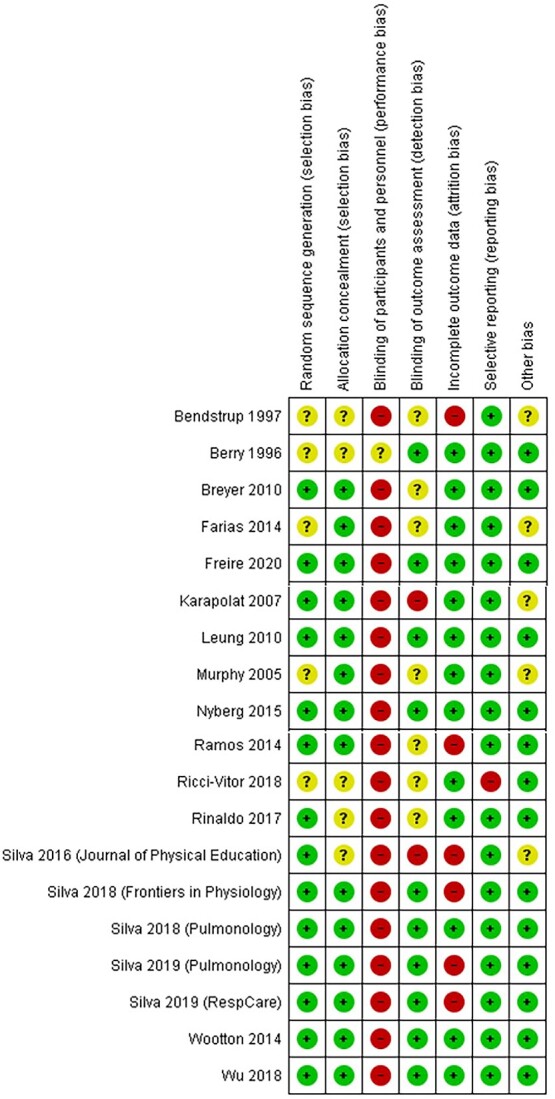
Risk of bias summary.

The risk of bias assessment is presented in Fig. 2. Overall, study quality was moderate, with most studies reporting allocation concealment and complete outcome data. Ten of the 19 studies reported assessor blinding.^16^^,^^28^^,^^30^^,^^32^^,^^34^^,^^36^^,^^37^^,^^39–41^ Blinding of participants and personnel was not feasible for this type of exercise intervention. Ten of the 19 studies used significance testing to confirm baseline comparability between treatment arms.^28–31^^,^^35–37^^,^^39–41^ Adjustment for baseline covariates when estimating the treatment effect was reported in 3 studies.^16^^,^^34^^,^^39^ Baseline comparability could not be confirmed in 1 study.^38^

### Effects on 6MWD

#### Minimal Equipment Programs Compared With Usual Care

**Figure 3:**
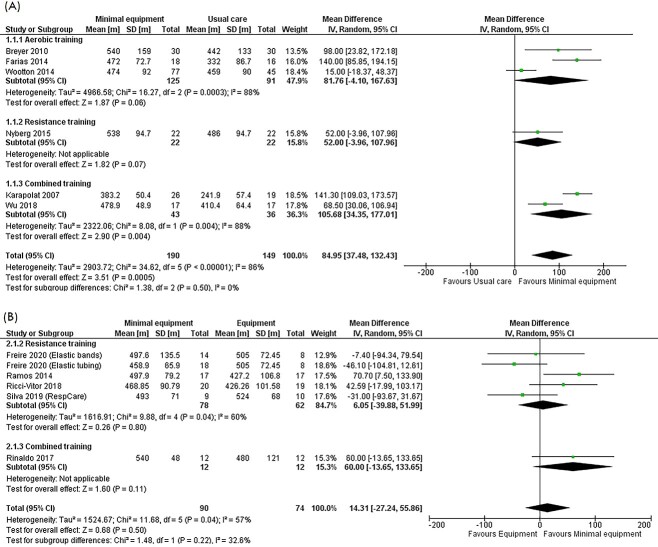
Meta-analysis of the effects of minimal equipment programs on post intervention 6-minute walk distance (6MWD). (A) Minimal equipment programs versus usual care. (B) Minimal equipment programs compared with equipment-based programs.

The meta-analysis included 56 RCTs in 339 participants, of which 3 studies performed aerobic training,^16^^,^^17^^,^^29^ 1 resistance training,^34^ and 2 combined aerobic and resistance training^31^^,^^41^ (Fig. 3A). Compared with usual care, minimal equipment programs improved 6MWD by 85 m (95% CI 37 to 132 m; moderate certainty). Subgroup analyses showed a larger effect size following combined training compared with aerobic or resistance training alone, but this was not statistically significant (Fig. 3A). There was high between-study heterogeneity (I^2^ = 86%).

Change scores for 6MWD were available for 1 study, which reported an improvement in 6MWD favoring the minimal equipment program (22 m, 95% CI = 6 to 39 m).^16^

#### Minimal Equipment Programs Compared With Exercise Equipment–Based Programs

Five RCTs in 164 participants, of which 4 studies performed resistance training^19^^,^^30^^,^^35^^,^^37^ and 1 combined training,^18^ were included in the meta-analysis (Fig. 3B). No difference in 6MWD was observed between minimal equipment and exercise equipment–based programs (14 m, 95% CI = −27 to 56 m; low certainty).

Change scores for 6MWD were reported in 2 studies, which were included in a sensitivity analysis.^19^^,^^30^ No differences in 6MWD were observed between the groups (7 m, 95% CI = −6 to 19 m, I^2^ = 0%) (Suppl. Appendix 2A).

### Effects on Secondary Outcomes

#### Minimal Equipment Programs Compared With Usual Care

##### Exercise capacity

Two RCTs in 151 participants reported the effects of minimal equipment training on ISWT distance compared with usual care.^16^^,^^33^ The pooled effect size was 168 m (95% CI = 19 to 317 m) favoring minimal equipment training (low certainty). One RCT in 125 participants reported a 241-second (95% CI = 133 to 349 seconds) improvement in ESWT time following an aerobic minimal equipment program.^16^

#### HRQoL

**Figure 4:**
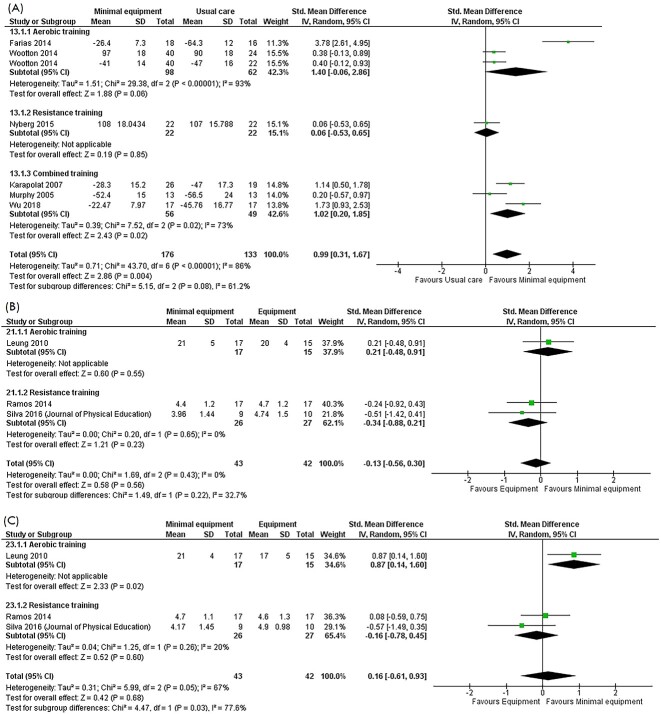
Meta-analysis of the effects of minimal equipment programs on postintervention disease-specific health-related quality of life (HRQoL). (A) Effects of minimal equipment programs versus usual care on disease-specific HRQoL total score. (B) Effects of minimal equipment programs versus equipment-based programs on Chronic Respiratory Disease Questionnaire (CRQ) dyspnea. (C) Effects of minimal equipment programs versus equipment-based programs on CRQ fatigue.

The meta-analysis included 56 RCTs in 309 participants, of which 2 studies performed aerobic training,^16^^,^^29^ 1 resistance training,^34^ and 3 combined training^31^^,^^33^^,^^41^ (Fig. 4A) compared with usual care. HRQoL was assessed using either the Chronic Respiratory Disease Questionnaire (CRQ) total score or St George’s Respiratory Questionnaire (SGRQ) total score, with 1 RCT reporting both outcomes.^16^ The pooled standardized mean difference was 0.99 (95% CI = 0.31 to 1.67) favoring minimal equipment training (moderate certainty).

Of the 5 RCTs that assessed HRQoL using the SGRQ,^16^^,^^29^^,^^31^^,^^33^^,^^41^ the pooled estimate for the SGRQ total score was −18.4 points (95% CI = −32.2 to −4.6 points) favoring minimal equipment training. Clinically significant improvements favoring minimal equipment were also observed for SGRQ symptoms score (−17.2 points, 95% CI = −34.2 to −0.2 points), activity score (−18.4 points, 95% CI = −34.6 to −2.1 points), and impact score (−16.2 points, 95% CI = −28.9 to −3.5 points) (see Suppl. Appendix S3 for meta-analyses). All meta-analyses were of moderate certainty.

Change scores for HRQoL measured by CRQ total score were reported in 2 studies.^16^^,^^34^ No differences in HRQoL were observed between the groups in the sensitivity analysis (−3.6 points, 95% CI = −4.0 to 11.2 points, I^2^ = 67%) (Suppl. Appendix 2B).

#### Peripheral Muscle Strength

One RCT in 44 participants evaluated the effects of a minimal equipment resistance program using elastic resistance bands compared with usual care strength on strength measured by electronic dynamometry.^34^ Significant between-group differences favoring minimal equipment were reported for shoulder flexor and knee extensor strength and upper limb functional capacity.

#### Dyspnea

One RCT in 129 participants^16^ and 1 RCT in 34 participants^29^ evaluated the effects of a minimal equipment aerobic program compared with usual care on dyspnea measured by the modified Medical Research Council (mMRC) dyspnea scale and the CRQ dyspnea domain, respectively. An improvement in mMRC dyspnea favoring minimal equipment was reported (−1.3 points, 95% CI = −1.8 to −0.8 points), but there were no between-group differences in CRQ dyspnea (0.2 points, 95% CI = −0.2 to 0.5 points).

#### Anxiety and Depression

Two RCTs in 76 and 71 participants compared the effects of an aerobic and a resistance minimal equipment training program, respectively, with usual care on anxiety and depression using the Hospital Anxiety and Depression Scale.^17^^,^^34^ No differences in Hospital Anxiety and Depression Scale anxiety (−2.2 points, 95% CI = −4.9 to 0.6 points; low certainty) or depression score (−2.9 points, 95% CI = −7.4 to 1.6 points; low certainty) were observed between groups (see Suppl. Appendix S4 for meta-analyses).

#### Minimal Equipment Programs Compared With Exercise Equipment–based Programs

##### Exercise capacity

One RCT in 32 participants evaluated the effects of a minimal equipment aerobic training program compared with an exercise equipment–based program.^32^ This study reported an improvement in ESWT time of 301 seconds (95% CI = 45 to 557 seconds) favoring minimal equipment training but no difference in ISWT distance between groups (39 m, 95% CI = −51 to 129 m).

#### HRQoL

Three RCTs in 85 participants, of which 1 study performed aerobic training^32^ and 2 resistance training,^19^^,^^38^ used the CRQ to compare the effects of minimal equipment training and exercise equipment–based training on HRQoL. No between-group differences were observed in the CRQ dyspnea (Fig. 4B), fatigue (Fig. 4C), emotional functioning (0.2 points, 95% CI = −0.5 to 0.9 points), or mastery (0.12 points, 95% CI = −0.6 to 0.9 points) domains. All meta-analyses were of low certainty. However, in the single RCT that compared walking training with cycle training, there was an improvement in CRQ total score by 15.0 points (95% CI = 4.0 to 26.0 points) favoring minimal equipment aerobic training.^32^

**Figure 5:**
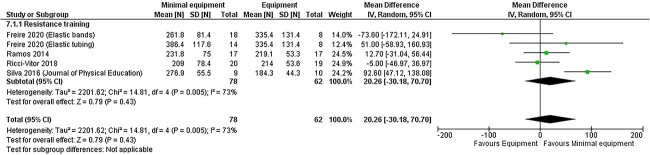
Meta-analysis of the effects of minimal equipment programs compared with equipment-based programs on postintervention knee extensor strength.

#### Peripheral Muscle Strength

Four RCTs in 140 participants compared the effects of minimal equipment resistance training using elastic bands and elastic tubing with exercise equipment–based training on strength measured by electronic dynamometry.^19^^,^^30^^,^^35^^,^^38^ No differences in improvements in strength were observed between groups for knee extensor strength (20 N, 95% CI = −30 to 71 N; low certainty) (Fig. 5) or strength of the shoulder flexors (6 N, 95% CI = −2 to 13 N; moderate certainty), shoulder abductors (3 N, 95% CI = −4 to 11 N; moderate certainty), elbow flexors (−6 N, 95% CI = −23 to 10 N; moderate certainty), or knee flexors (−30 N, 95% CI = −78 to 19 N; low certainty) (see Suppl. Appendix S5 for meta-analyses).

Change scores for strength were reported in 2 studies.^19^^,^^30^ No differences in lower limb strength (−14.7 N, 95% CI = −36.4 to 7.0 N, I^2^ = 0%) or upper limb strength (0.7 N, 95% CI = −7.4 to 8.8 N, I^2^ = 0%) were observed between groups in the sensitivity analysis (Suppl. Appendix 2C and 2D).

## Discussion

This meta-analysis provides moderate-quality evidence that in people with COPD, pulmonary rehabilitation programs using minimal equipment for aerobic and resistance training led to large, clinically relevant improvements in exercise capacity and HRQoL. When compared with exercise equipment–based pulmonary rehabilitation programs, there were no significant differences between improvements in exercise capacity, upper and lower limb strength, HRQoL, or dyspnea, indicating that minimal equipment programs may be a suitable alternative in settings where access to exercise equipment is limited.

Compared with usual care, the improvement in 6MWD following minimal equipment training exceeded the minimal clinically important difference (MCID) of 30 m (95% CI = 25 to 33 m)^42^ and was greater in magnitude than the effect reported by McCarthy and colleagues (44 m, 95% CI = 33 to 55 m; 1879 participants; 38 studies),^1^ which included both minimal equipment and exercise equipment–based pulmonary rehabilitation programs. Improvements in ISWT distance also exceeded the proposed MCID of 35 m^43^ and were larger than in previously reported studies (MD 40 m, 95% CI 22 to 57 m; 694 participants; 8 studies).^1^ In addition, improvement in ESWT time from a single RCT exceeded the lower end of the MCID of 174 seconds.^44^ These results suggest that the key principles of minimal equipment aerobic training to obtain physiological benefit are (1) individually tailored prescription of exercise intensity based on initial field walking tests or moderate to somewhat severe levels of dyspnea, and (2) supervision by experienced clinicians to ensure appropriate progression of training intensity and duration over the course of a pulmonary rehabilitation program. Ground walking training, the most common minimal equipment training modality for aerobic training, can achieve physiological changes in skeletal muscle when prescribed at walking at 80% of average 6-minute walk test speed or 70% peak ISWT speed, which exceeds an intensity of ≥50% VO_2_peak.^45^^,^^46^

Clinically significant improvements in HRQoL were also observed following minimal equipment training compared with usual care. There was a large pooled effect size for disease-specific HRQoL total score, and improvements in SGRQ total score and all SGRQ domains exceeded the MCID of −4 points.^47^ Less consistent benefits were observed for dyspnea, with improvements in mMRC dyspnea that exceeded the proposed MCID of −1 point^48^ but small, nonsignificant improvements in the CRQ dyspnea domain in individual RCTs. These findings should be interpreted with caution, however, because a lack of common outcome measures evaluating the same constructs of dyspnea (ie, severity of dyspnea as opposed to impact of dyspnea on HRQoL) across studies prevented the pooling of results in a meta-analysis. It remains unclear whether minimal equipment programs can elicit clinically meaningful improvements in dyspnea in people with COPD. Dyspnea-specific outcome measures should be used to evaluate the effects of minimal equipment exercise training on dyspnea in future studies.

No significant differences in improvements in 6MWD, ISWT distance, upper or lower limb strength, or any of the CRQ domains were found between the minimal equipment and exercise equipment–based training groups in this review. These findings are consistent with a previous UK study that showed that supervised pulmonary rehabilitation using minimal equipment can produce clinically significant benefits in ISWT distance (within-group change = 57 m, 95% CI = 48 to 65 m) and in all CRQ domains that were noninferior to those observed in matched participants undergoing conventional pulmonary rehabilitation.^20^ This provides reassuring evidence that resistance training programs using minimal equipment and supervised by experienced clinicians can achieve similar improvements in exercise capacity, strength, HRQoL, and dyspnea to conventional programs well-resourced with gymnasium equipment.

The current review also supports supervised resistance training with elastic bands/tubing as a substitute for exercise machine resistance training in people with COPD. Elastic resistance bands/tubing have the advantage of transferring more easily to the home environment compared with weight machines, thus promoting continued exercise and maintenance of benefits. Qualitative studies in people with COPD have demonstrated high satisfaction with minimal equipment training programs in home-based and telerehabilitation settings, with participants reporting that the minimal equipment training modalities were easy and more convenient to use than exercise equipment at hospital-based pulmonary rehabilitation programs and required minimal space to set up in the home environment.^49^^,^^50^ Poor mobility, lack of transport, cost of travel, and poor weather have consistently been reported as barriers to both uptake and completion of center-based pulmonary rehabilitation programs.^51^^,^^52^ The convenience and transferability of minimal equipment training programs to home and telerehabilitation settings may increase uptake of and adherence to pulmonary rehabilitation, particularly for people with COPD with impaired mobility or from low socioeconomic backgrounds.

Qualitative studies also highlight the importance of staff skill set for facilitating adherence to and completion of pulmonary rehabilitation. In a survey of 74 multidisciplinary pulmonary rehabilitation specialists in the United Kingdom, 96% of participants agreed that the way health care professionals deliver rehabilitation has an important influence on the success of the program.^53^ Adequate supervision and support from experienced clinicians may be more influential than access to specialist exercise equipment for improving outcomes and increasing the confidence and readiness of people with COPD to engage in longer-term exercise and physical activity.^54^ Patient perceptions of the importance of regular monitoring and support from health care professionals to promote and maintain motivation is supported by telerehabilitation studies using minimal equipment.^50^^,^^55^

### Limitations

The major limitation of this systematic review is the small number of RCTs that directly compared pulmonary rehabilitation using minimal equipment with exercise equipment–based pulmonary rehabilitation to include in the meta-analysis. The small number of included studies also limited the ability to compare minimal equipment resistance training with usual care, and the ability to conduct subgroup analyses to explore the effects of specific modalities of minimal equipment training (eg, indoor vs outdoor walking, elastic resistance bands vs body weight resistance exercises) and the impact of severity of airflow limitation and cardiorespiratory fitness levels at baseline on outcomes.

Another limitation of this review is the substantial statistical heterogeneity evident in the meta-analyses. Although there was no variation in the direction of effect of minimal equipment programs compared with usual care on 6MWD and HRQoL in the main and sensitivity analyses, the substantial statistical heterogeneity affects the extent to which the findings of this review can be generalized to the wider population of COPD. The statistical heterogeneity in the meta-analyses may be attributed to clinical diversity across the included studies. There was anticipated variability in the minimal equipment training programs with respect to type and modality of training. Variability was also detected in the length of the training program and in the progression and monitoring of exercise intensity across studies. Because less than 10 studies could be pooled in the meta-analyses, there is much uncertainty in measures of heterogeneity such as the I^2^ statistic when there are few included studies.^25^

Substantial statistical heterogeneity in the meta-analyses has previously been detected by Cochrane systematic reviews evaluating the effects of pulmonary rehabilitation in people with COPD. McCarthy and colleagues reported substantial heterogeneity across studies for 6MWD (I^2^ = 74%) and for HRQoL measured by the CRQ and SGRQ (I^2^ = 64%) following completion of a comprehensive pulmonary rehabilitation program.^1^ Variations in the content of the intervention program, setting of the program, and severity of COPD were hypothesized to contribute to the substantial heterogeneity within the meta-analyses. Subsequently, the quality of the evidence provided by this review was downgraded due to substantial statistical heterogeneity. The certainty of evidence was downgraded to moderate in the comparison of minimal equipment training with usual care and to low in the comparison with equipment-based training due to risk of selection, detection, and attrition bias; and due to imprecision related to relatively few participants in some meta-analyses resulting in wide CIs around the estimate of effect.

However, there was relatively low methodological diversity across the included studies with respect to study design and outcome measurement tools. All included studies were RCTs conducted in hospital- or university-based outpatient settings and used standardized pulmonary rehabilitation assessment tools for functional exercise capacity, strength, and HRQoL. Studies were excluded from the review if they did not provide exercise training at an intensity and duration known to produce skeletal muscle adaptations. We are confident that pooling these data has improved our understanding of whether minimal equipment programs improve functional exercise capacity and HRQoL in people with symptomatic COPD compared with usual care, but whether minimal equipment programs can provide the same benefits as programs with access to exercise equipment should be interpreted with caution.

Pulmonary rehabilitation programs using minimal equipment can achieve clinically significant improvements in exercise capacity, strength, and HRQoL in people with COPD and are comparable with exercise equipment–based programs when supervised by experienced clinicians and performed at the training intensity and duration recommended by clinical practice guidelines. Limited access to hospital-based gymnasiums need not be a barrier to establishing effective exercise training programs and increasing access to pulmonary rehabilitation to a wider group of people with COPD, particularly in rural and remote areas and low-middle-income countries.

## Supplementary Material

PTJ-Data-supplement-R1mkl_pzad013

## Data Availability

Data are available from the corresponding author on reasonable request.

## Systematic Review Registration

This systematic review was prospectively registered at PROSPERO (www.crd.york.ac.uk/PROSPERO): CRD42021048159.
