# Supplementary material for: Pulmonary Rehabilitation Using Minimal Equipment for People With Chronic Obstructive Pulmonary Disease: A Systematic Review and Meta-Analysis
Source: Phys Ther. 2023 Feb 9;103(5):pzad013. doi: 10.1093/ptj/pzad013 (PMC10158642; doi:10.1093/ptj/pzad013)

Supplementary Appendix S1 Search strategy

#1 MeSH DESCRIPTOR Pulmonary Disease, Chronic Obstructive Explode All
#2 MeSH DESCRIPTOR Bronchitis, Chronic
#3 (obstruct*) near3 (pulmonary or lung* or airway* or airflow* or bronch* or respirat*)
#4 COPD:MISC1
#5 (COPD OR COAD OR COBD OR AECOPD):TI,AB,KW
#6 #1 OR #2 OR #3 OR #4 OR #5
#7 MeSH DESCRIPTOR Rehabilitation Explode All
#8 MeSH DESCRIPTOR Respiratory Therapy Explode All
#9 MeSH DESCRIPTOR Physical Therapy Modalities Explode All
#10 rehabilitat* or fitness* or exercis* or train* or physiotherap* or (physical* NEXT therap*)
#11 #7 or #8 or #9 or #10
#12 #11 AND #4

Supplementary Appendix S2 Meta-analysis of the effects of minimal equipment programmes compared with usual care and with equipment-based programmes using change scores

a Compared with equipment-based programmes: Change in six-minute walk distance

	Minimal equipment			Equipment				Mean Difference	Mean Difference
Study or Subgroup	Mean [Metres]	SD [Metres]	Total	Mean [Metres]	SD [Metres]	Total	Weight	IV, Random, 95% CI	IV, Random, 95% CI
**32.2.1 Resistance training**									
Freire 2020 (Elastic bands)	15	38.103	14	2	19.1383	8	27.5%	13.00 [-10.96, 36.96]	
Freire 2020 (Elastic tubing)	3	18.0982	18	2	19.1383	8	64.1%	1.00 [-14.68, 16.68]	
Ramos 2014	73	70.0181	17	42	58.3484	17	8.4%	31.00 [-12.33, 74.33]	
**Subtotal (95% CI)**			**49**			**33**	**100.0%**	**6.81 [-5.74, 19.37]**	
Heterogeneity: Tau² = 0.00; Chi² = 1.98, df = 2 (P = 0.37); I² = 0%									
Test for overall effect: Z = 1.06 (P = 0.29)									
**Total (95% CI)**			**49**			**33**	**100.0%**	**6.81 [-5.74, 19.37]**	
Heterogeneity: Tau² = 0.00; Chi² = 1.98, df = 2 (P = 0.37); I² = 0%									
Test for overall effect: Z = 1.06 (P = 0.29)									
Test for subgroup differences: Not applicable									

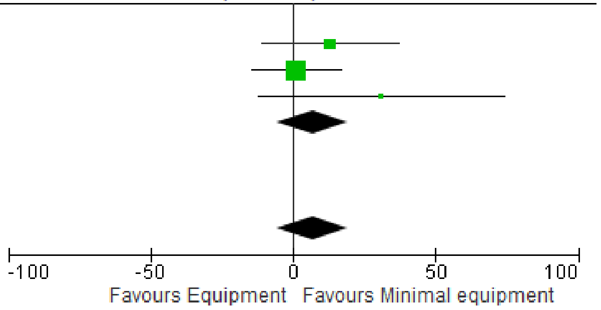

b Compared with usual care: Change in health-related quality of life measured by Chronic Respiratory Disease Questionnaire total score

	Minimal equipment			Usual care				Mean Difference
**Study or Subgroup**	**Mean [Points]**	**SD [Points]**	**Total**	**Mean [Points]**	**SD [Points]**	**Total**	**Weight**	**IV, Random, 95% CI**
**32.1.1 Aerobic training**								
Wootton 2014	8	13.5674	81	1	13.7755	48	56.4%	7.00 [2.11, 11.89]
**Subtotal (95% CI)**			**81**			**48**	**56.4%**	**7.00 [2.11, 11.89]**
Heterogeneity: Not applicable								
Test for overall effect: Z = 2.81 (P = 0.005)								
**32.1.2 Resistance training**								
Nyberg 2015	1.6	12.6304	22	2.4	12.1793	22	43.6%	-0.80 [-8.13, 6.53]
**Subtotal (95% CI)**			**22**			**22**	**43.6%**	**-0.80 [-8.13, 6.53]**
Heterogeneity: Not applicable								
Test for overall effect: Z = 0.21 (P = 0.83)								
**Total (95% CI)**			**103**			**70**	**100.0%**	**3.60 [-3.98, 11.18]**
Heterogeneity: Tau² = 20.31; Chi² = 3.01, df = 1 (P = 0.08); I² = 67%								
Test for overall effect: Z = 0.93 (P = 0.35)								
Test for subgroup differences: Chi² = 3.01, df = 1 (P = 0.08), I² = 66.8%								

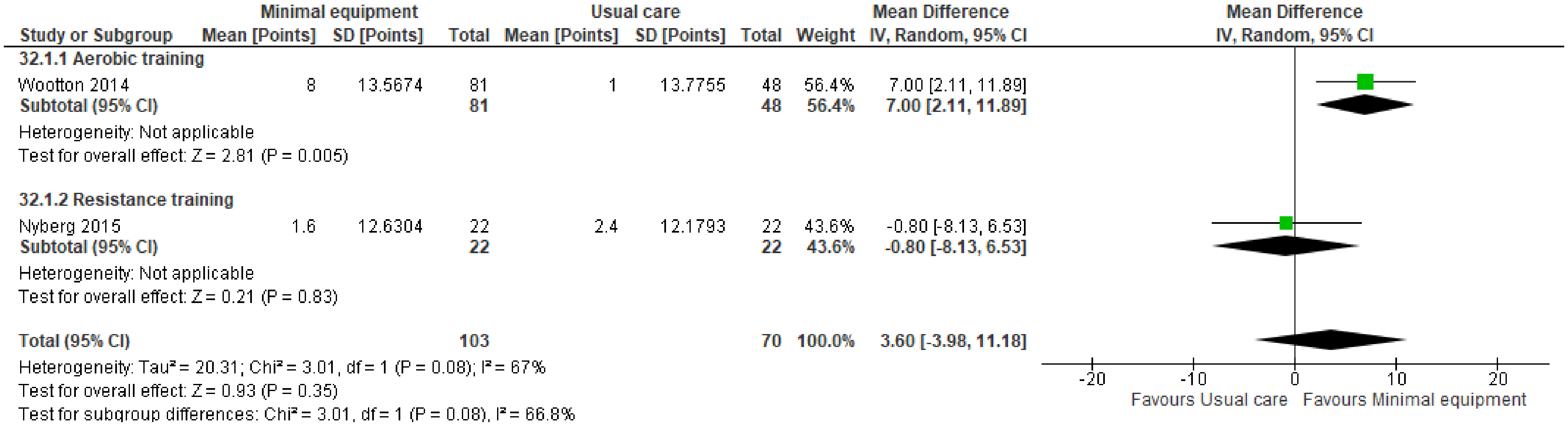

c Compared with equipment-based programmes: Change in knee extension strength

	Minimal equipment			Equipment				Mean Difference
**Study or Subgroup**	**Mean [N]**	**SD [N]**	**Total**	**Mean [N]**	**SD [N]**	**Total**	**Weight**	**IV, Random, 95% CI**
**32.3.1 Resistance training**								
Freire 2020 (Elastic bands)	56.88	99.3622	14	50.01	51.6135	8	11.8%	6.87 [-56.28, 70.02]
Freire 2020 (Elastic tubing)	30.6	83.2314	18	50.01	51.6135	8	17.1%	-19.41 [-71.92, 33.10]
Ramos 2014	17	46.6788	17	34.1	27.4238	17	71.1%	-17.10 [-42.84, 8.64]
**Subtotal (95% CI)**			**49**			**33**	**100.0%**	**-14.66 [-36.37, 7.04]**
Heterogeneity: Tau² = 0.00; Chi² = 0.51, df = 2 (P = 0.77); I² = 0%								
Test for overall effect: Z = 1.32 (P = 0.19)								
**Total (95% CI)**			**49**			**33**	**100.0%**	**-14.66 [-36.37, 7.04]**
Heterogeneity: Tau² = 0.00; Chi² = 0.51, df = 2 (P = 0.77); I² = 0%								
Test for overall effect: Z = 1.32 (P = 0.19)								
Test for subgroup differences: Not applicable								

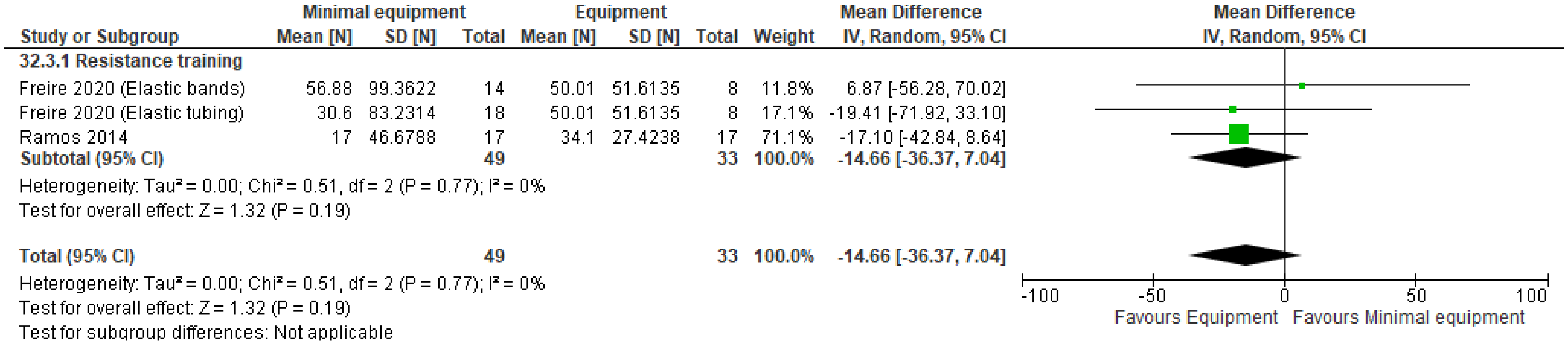

d Compared with equipment-based programmes: Change in shoulder flexion strength

	Minimal equipment			Equipment				Mean Difference
**Study or Subgroup**	**Mean [N]**	**SD [N]**	**Total**	**Mean [N]**	**SD [N]**	**Total**	**Weight**	**IV, Random, 95% CI**
**32.4.1 Resistance training**								
Freire 2020 (Elastic bands)	12.75	33.9636	14	4.9	17.5953	8	14.1%	7.85 [-13.72, 29.42]
Freire 2020 (Elastic tubing)	4.9	31.5511	18	4.9	17.5953	8	18.2%	0.00 [-19.00, 19.00]
Ramos 2014	9.6	17.1155	17	10.2	11.6697	17	67.7%	-0.60 [-10.45, 9.25]
**Subtotal (95% CI)**			**49**			**33**	**100.0%**	**0.70 [-7.40, 8.80]**
Heterogeneity: Tau² = 0.00; Chi² = 0.49, df = 2 (P = 0.78); I² = 0%								
Test for overall effect: Z = 0.17 (P = 0.87)								
**Total (95% CI)**			**49**			**33**	**100.0%**	**0.70 [-7.40, 8.80]**
Heterogeneity: Tau² = 0.00; Chi² = 0.49, df = 2 (P = 0.78); I² = 0%								
Test for overall effect: Z = 0.17 (P = 0.87)								
Test for subgroup differences: Not applicable								

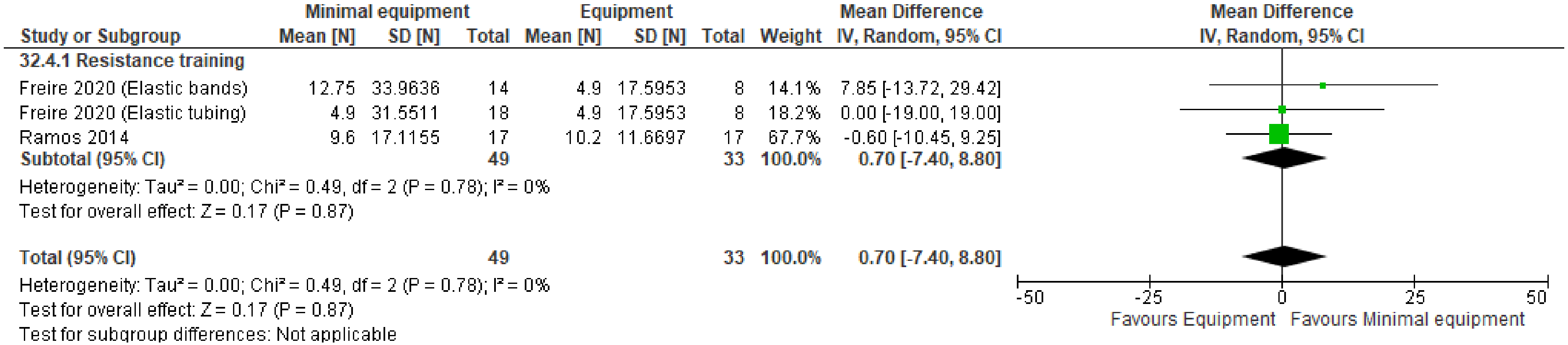

Supplementary Appendix S3 Meta-analysis of the effects of minimal equipment programmes compared with usual care on health-related quality of life measured by the St George's Respiratory Questionnaire

a Total score

	Minimal equipment			Usual care				Mean Difference
**Study or Subgroup**	**Mean [Points]**	**SD [Points]**	**Total**	**Mean [Points]**	**SD [Points]**	**Total**	**Weight**	**IV, Random, 95% CI**
**Aerobic training**								
Farias 2014	26.4	7.3	18	64.3	12	16	21.0%	-37.90 [-44.68, -31.12]
Wootton 2014	41	14	80	47	16	45	21.4%	-6.00 [-11.59, -0.41]
**Subtotal (95% CI)**			**98**			**61**	**42.4%**	**-21.89 [-53.15, 9.37]**
Heterogeneity: Tau² = 498.76; Chi² = 50.63, df = 1 (P < 0.00001); I² = 98%								
Test for overall effect: Z = 1.37 (P = 0.17)								
**Combined training**								
Karapolat 2007	28.3	15.2	26	47	17.3	19	19.9%	-18.70 [-28.43, -8.97]
Murphy 2005	52.4	15	13	56.5	24	13	17.4%	-4.10 [-19.48, 11.28]
Wu 2018	22.47	7.97	17	45.76	16.77	17	20.3%	-23.29 [-32.12, -14.46]
**Subtotal (95% CI)**			**56**			**49**	**57.6%**	**-17.12 [-26.58, -7.67]**
Heterogeneity: Tau² = 38.30; Chi² = 4.50, df = 2 (P = 0.11); I² = 56%								
Test for overall effect: Z = 3.55 (P = 0.0004)								
**Total (95% CI)**			**154**			**110**	**100.0%**	**-18.42 [-32.23, -4.61]**
Heterogeneity: Tau² = 224.21; Chi² = 55.13, df = 4 (P < 0.00001); I² = 93%								
Test for overall effect: Z = 2.61 (P = 0.009)								
Test for subgroup differences: Chi² = 0.08, df = 1 (P = 0.77), I² = 0%								

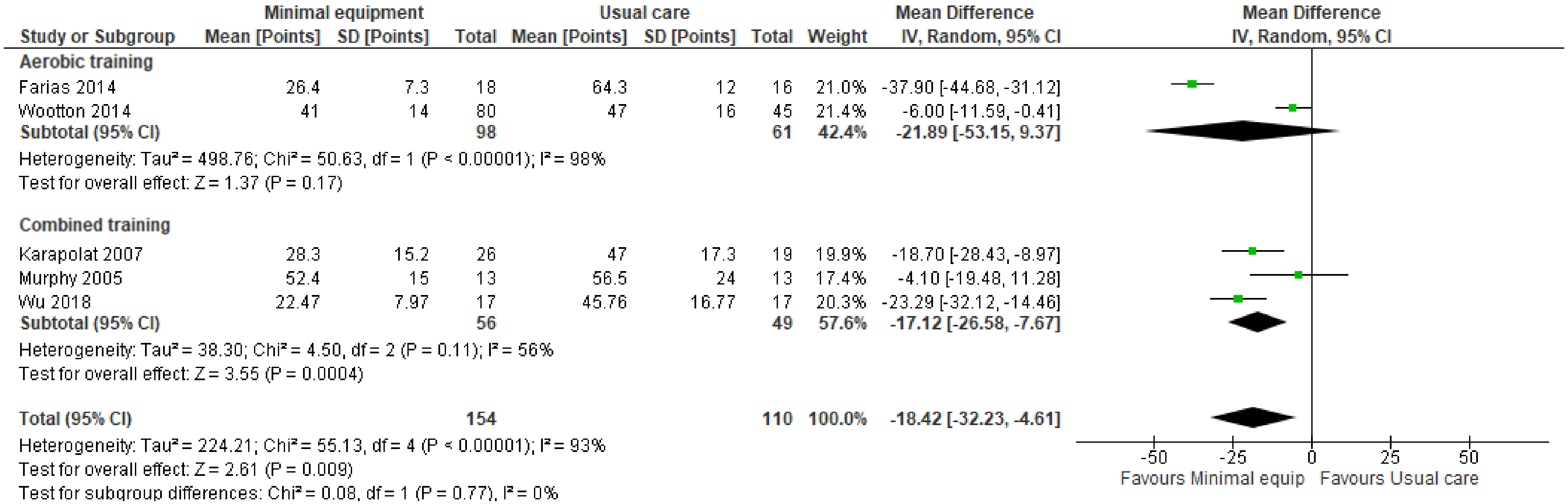

b Symptoms score

	Minimal equipment			Usual care				Mean Difference
Study or Subgroup	Mean [Points]	SD [Points]	Total	Mean [Points]	SD [Points]	Total	Weight	IV, Random, 95% CI
**Aerobic training**								
Farias 2014	25	12.5	18	61	14.1	16	20.4%	-36.00 [-45.00, -27.00]
Wootton 2014	50	23	80	59	21	45	20.6%	-9.00 [-16.94, -1.06]
**Subtotal (95% CI)**			**98**			**61**	**41.0%**	**-22.41 [-48.87, 4.05]**
Heterogeneity: Tau² = 345.74; Chi² = 19.43, df = 1 (P < 0.0001); I² = 95%								
Test for overall effect: Z = 1.66 (P = 0.10)								
**Combined training**								
Karapolat 2007	37.5	16.3	26	45.9	24.7	19	19.3%	-8.40 [-21.15, 4.35]
Murphy 2005	74	9.2	13	67.2	17.3	13	19.9%	6.80 [-3.85, 17.45]
Wu 2018	20.82	10.64	17	59.88	20.32	17	19.8%	-39.06 [-49.96, -28.16]
**Subtotal (95% CI)**			**56**			**49**	**59.0%**	**-13.57 [-41.36, 14.22]**
Heterogeneity: Tau² = 568.95; Chi² = 35.67, df = 2 (P < 0.00001); I² = 94%								
Test for overall effect: Z = 0.96 (P = 0.34)								
**Total (95% CI)**			**154**			**110**	**100.0%**	**-17.20 [-34.18, -0.22]**
Heterogeneity: Tau² = 347.60; Chi² = 57.59, df = 4 (P < 0.00001); I² = 93%								
Test for overall effect: Z = 1.98 (P = 0.05)								
Test for subgroup differences: Chi² = 0.20, df = 1 (P = 0.65), I² = 0%								

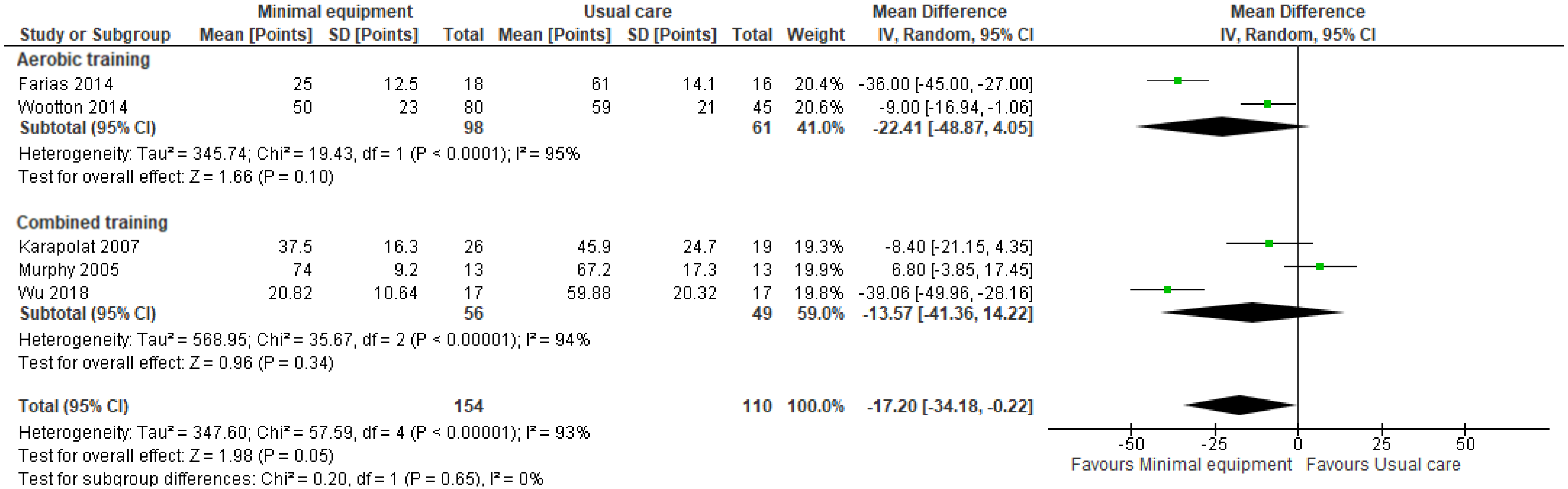

c Activity score

Study or Subgroup	Minimal equipment Mean [Points]	SD [Points]	Total	Usual care Mean [Points]	SD [Points]	Total	Weight	Mean Difference IV, Random, 95% CI
**Aerobic training**								
Farias 2014	36.5	9	18	76.5	11.7	16	21.8%	-40.00 [-47.08, -32.92]
Wootton 2014	59	18	80	66	20	45	21.8%	-7.00 [-14.05, 0.05]
**Subtotal (95% CI)**			**98**			**61**	**43.5%**	**-23.50 [-55.84, 8.84]**
Heterogeneity: Tau² = 531.50; Chi² = 41.89, df = 1 (P < 0.00001); I² = 98%								
Test for overall effect: Z = 1.42 (P = 0.15)								
**Combined training**								
Karapolat 2007	42.5	22.1	26	66.7	24	19	19.5%	-24.20 [-37.93, -10.47]
Murphy 2005	55.7	23	13	52.4	29	13	16.9%	3.30 [-16.82, 23.42]
Wu 2018	33	9.11	17	52.71	24.43	17	20.1%	-19.71 [-32.10, -7.32]
**Subtotal (95% CI)**			**56**			**49**	**56.5%**	**-15.42 [-29.36, -1.48]**
Heterogeneity: Tau² = 91.68; Chi² = 5.14, df = 2 (P = 0.08); I² = 61%								
Test for overall effect: Z = 2.17 (P = 0.03)								
**Total (95% CI)**			**154**			**110**	**100.0%**	**-18.36 [-34.63, -2.09]**
Heterogeneity: Tau² = 303.56; Chi² = 48.50, df = 4 (P < 0.00001); I² = 92%								
Test for overall effect: Z = 2.21 (P = 0.03)								
Test for subgroup differences: Chi² = 0.20, df = 1 (P = 0.65), I² = 0%								

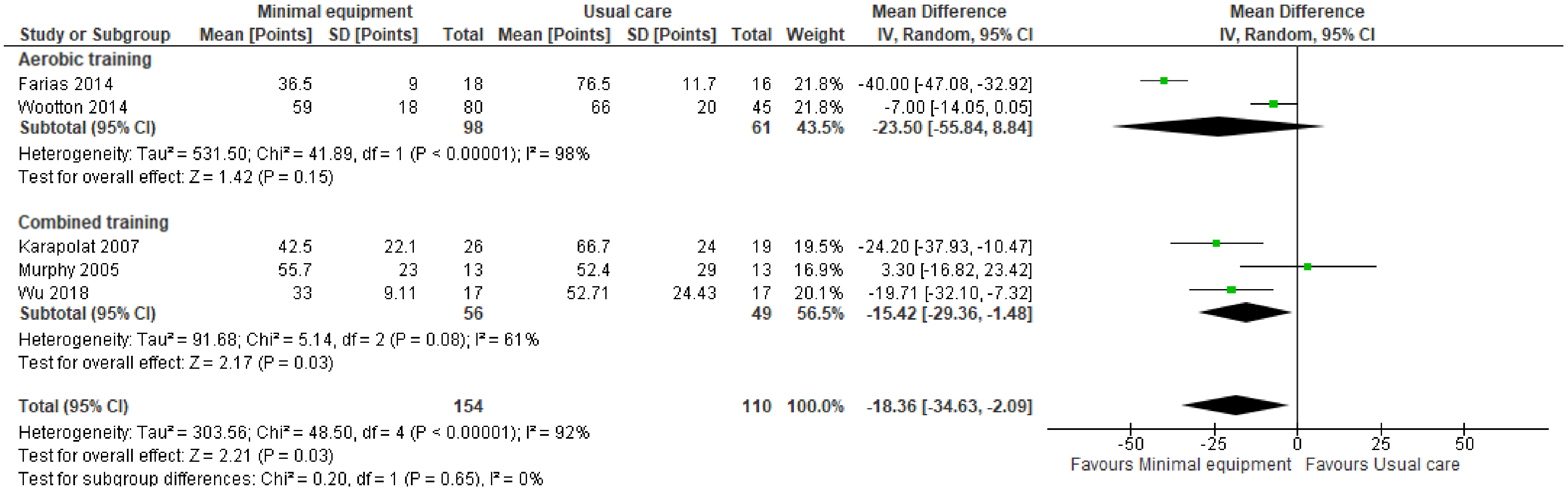

d Impact score

Study or Subgroup	Minimal equipment Mean [Points]	Minimal equipment SD [Points]	Minimal equipment Total	Usual care Mean [Points]	Usual care SD [Points]	Usual care Total	Weight	Mean Difference IV, Random, 95% CI
**Aerobic training**								
Farias 2014	21.1	7.8	18	58.2	17.3	16	20.5%	-37.10 [-46.31, -27.89]
Wootton 2014	27	14	80	33	16	45	22.0%	-6.00 [-11.59, -0.41]
**Subtotal (95% CI)**			**98**			**61**	**42.5%**	**-21.33 [-51.80, 9.15]**
Heterogeneity: Tau² = 468.49; Chi² = 32.00, df = 1 (P < 0.00001); I² = 97%								
Test for overall effect: Z = 1.37 (P = 0.17)								
**Combined training**								
Karapolat 2007	17.2	15.1	26	33.4	16.8	19	20.4%	-16.20 [-25.73, -6.67]
Murphy 2005	43.7	14	13	41.7	25.6	13	16.9%	2.00 [-13.86, 17.86]
Wu 2018	16.65	10.99	17	37.94	17.68	17	20.2%	-21.29 [-31.19, -11.39]
**Subtotal (95% CI)**			**56**			**49**	**57.5%**	**-13.40 [-24.82, -1.98]**
Heterogeneity: Tau² = 66.83; Chi² = 6.01, df = 2 (P = 0.05); I² = 67%								
Test for overall effect: Z = 2.30 (P = 0.02)								
**Total (95% CI)**			**154**			**110**	**100.0%**	**-16.19 [-28.88, -3.49]**
Heterogeneity: Tau² = 182.40; Chi² = 38.08, df = 4 (P < 0.00001); I² = 89%								
Test for overall effect: Z = 2.50 (P = 0.01)								
Test for subgroup differences: Chi² = 0.23, df = 1 (P = 0.63), I² = 0%								

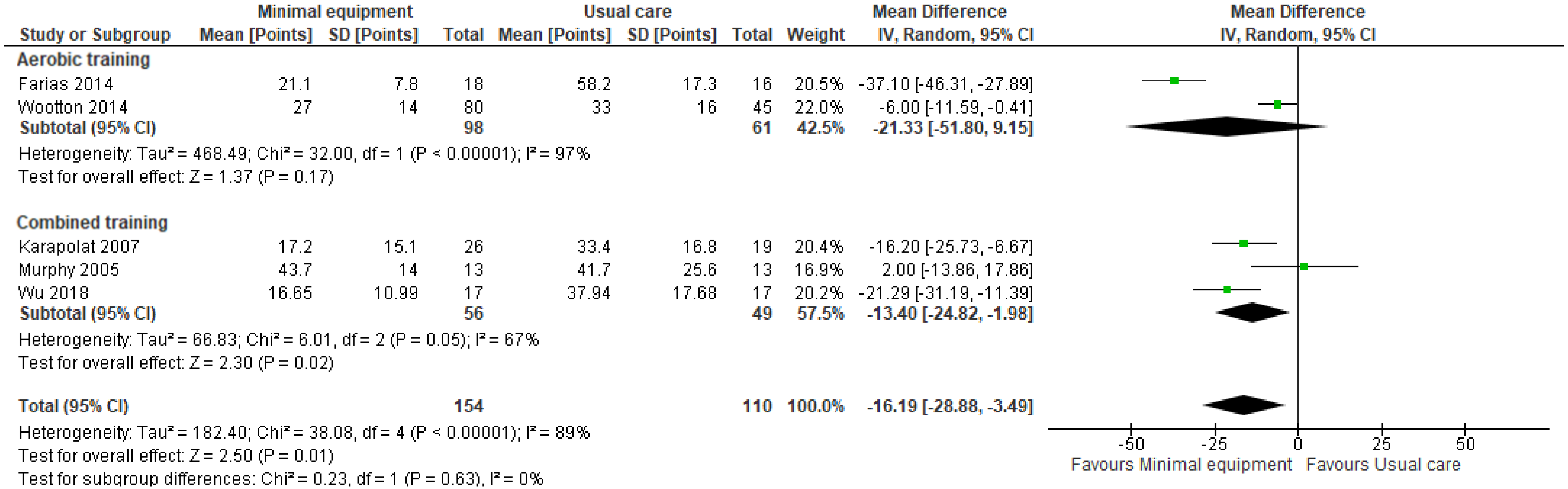

Supplementary Appendix S4 Meta-analysis of the effects of minimal equipment programmes compared with usual care on anxiety and depression measured by the Hospital Anxiety and Depression Scale

a Anxiety

	Minimal equipment			Usual care				Mean Difference
**Study or Subgroup**	**Mean [Points]**	**SD [Points]**	**Total**	**Mean [Points]**	**SD [Points]**	**Total**	**Weight**	**IV, Random, 95% CI**
**Aerobic training**								
Breyer 2010	6.6	2.3	15	10.2	3.6	17	49.2%	-3.60 [-5.67, -1.53]
**Subtotal (95% CI)**			**15**			**17**	**49.2%**	**-3.60 [-5.67, -1.53]**
Heterogeneity: Not applicable								
Test for overall effect: Z = 3.41 (P = 0.0007)								
**Resistance training**								
Nyberg 2015	3.6	3.2	22	4.4	3.4	22	50.8%	-0.80 [-2.75, 1.15]
**Subtotal (95% CI)**			**22**			**22**	**50.8%**	**-0.80 [-2.75, 1.15]**
Heterogeneity: Not applicable								
Test for overall effect: Z = 0.80 (P = 0.42)								
**Total (95% CI)**			**37**			**39**	**100.0%**	**-2.18 [-4.92, 0.57]**

Heterogeneity: Tau² = 2.87; Chi² = 3.72, df = 1 (P = 0.05); I² = 73%
Test for overall effect: Z = 1.56 (P = 0.12)
Test for subgroup differences: Chi² = 3.72, df = 1 (P = 0.05), I² = 73.1%

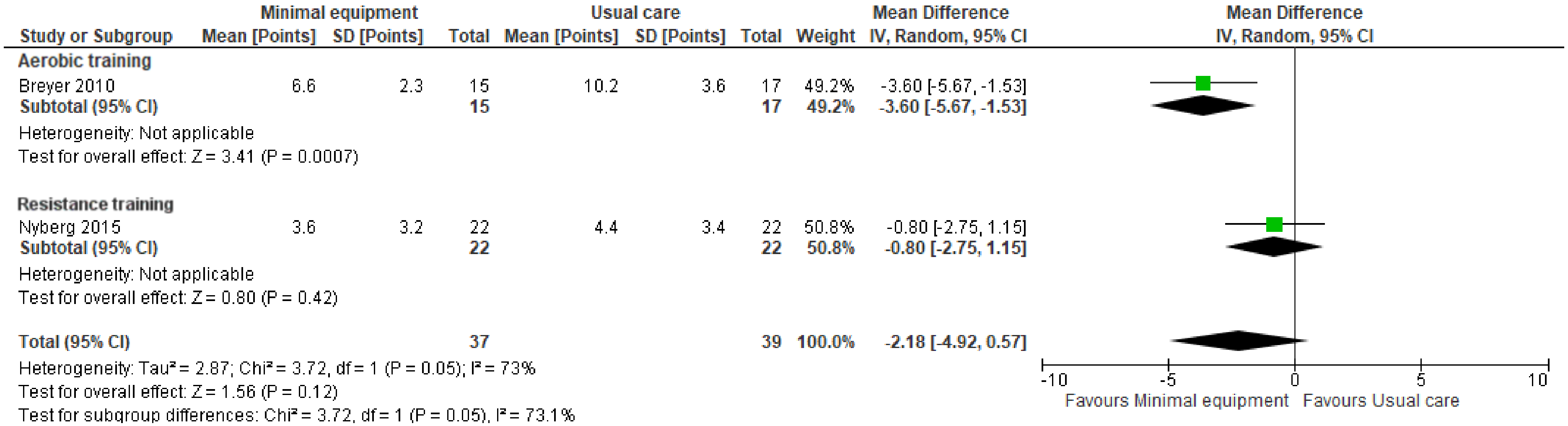

b Depression

	Minimal equipment			Usual care				Mean Difference
Study or Subgroup	Mean [Points]	SD [Points]	Total	Mean [Points]	SD [Points]	Total	Weight	IV, Random, 95% CI
**Aerobic training**								
Breyer 2010	6.3	3	12	11.6	3.2	15	47.9%	-5.30 [-7.65, -2.95]
**Subtotal (95% CI)**			**12**			**15**	**47.9%**	**-5.30 [-7.65, -2.95]**
Heterogeneity: Not applicable								
Test for overall effect: Z = 4.43 (P < 0.00001)								
**Resistance training**								
Nyberg 2015	2.8	2.4	22	3.5	2.5	22	52.1%	-0.70 [-2.15, 0.75]
**Subtotal (95% CI)**			**22**			**22**	**52.1%**	**-0.70 [-2.15, 0.75]**
Heterogeneity: Not applicable								
Test for overall effect: Z = 0.95 (P = 0.34)								
**Total (95% CI)**			**34**			**37**	**100.0%**	**-2.90 [-7.41, 1.60]**

Heterogeneity: $Tau^2 = 9.59$; $Chi^2 = 10.69$, df = 1 (P = 0.001); $I^2 = 91\%$
Test for overall effect: Z = 1.26 (P = 0.21)
Test for subgroup differences: $Chi^2 = 10.69$, df = 1 (P = 0.001), $I^2 = 90.6\%$

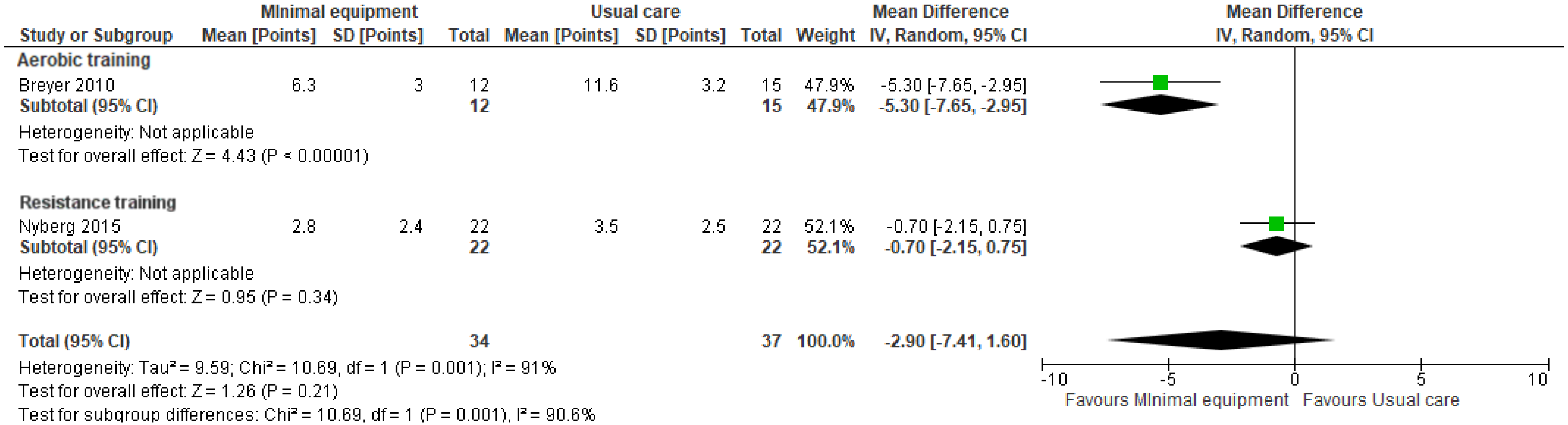

Supplementary Appendix S5 Meta-analysis of the effects of minimal equipment programmes compared with equipment-based programmes on post intervention strength

a Shoulder flexion strength

Study or Subgroup	Minimal equipment Mean [N]	SD [N]	Total	Equipment Mean [N]	SD [N]	Total	Weight	Mean Difference IV, Random, 95% CI
**Resistance training**								
Freire 2020 (Elastic bands)	109.8	44.1	14	83.4	36.3	8	5.2%	26.40 [-7.75, 60.55]
Freire 2020 (Elastic tubing)	72.6	29.4	18	83.4	36.3	8	7.5%	-10.80 [-39.39, 17.79]
Ramos 2014	66.8	22.6	17	62.2	19.5	17	30.3%	4.60 [-9.59, 18.79]
Ricci-Vitor 2018	64	23.7	20	61.8	19.4	19	33.1%	2.20 [-11.36, 15.76]
Silva 2016	89.9	17.4	9	78	18.1	10	23.9%	11.90 [-4.07, 27.87]
**Subtotal (95% CI)**			**78**			**62**	**100.0%**	**5.54 [-2.27, 13.35]**
Heterogeneity: Tau² = 0.00; Chi² = 3.55, df = 4 (P = 0.47); I² = 0%								
Test for overall effect: Z = 1.39 (P = 0.16)								
**Total (95% CI)**			**78**			**62**	**100.0%**	**5.54 [-2.27, 13.35]**
Heterogeneity: Tau² = 0.00; Chi² = 3.55, df = 4 (P = 0.47); I² = 0%								
Test for overall effect: Z = 1.39 (P = 0.16)								
Test for subgroup differences: Not applicable								

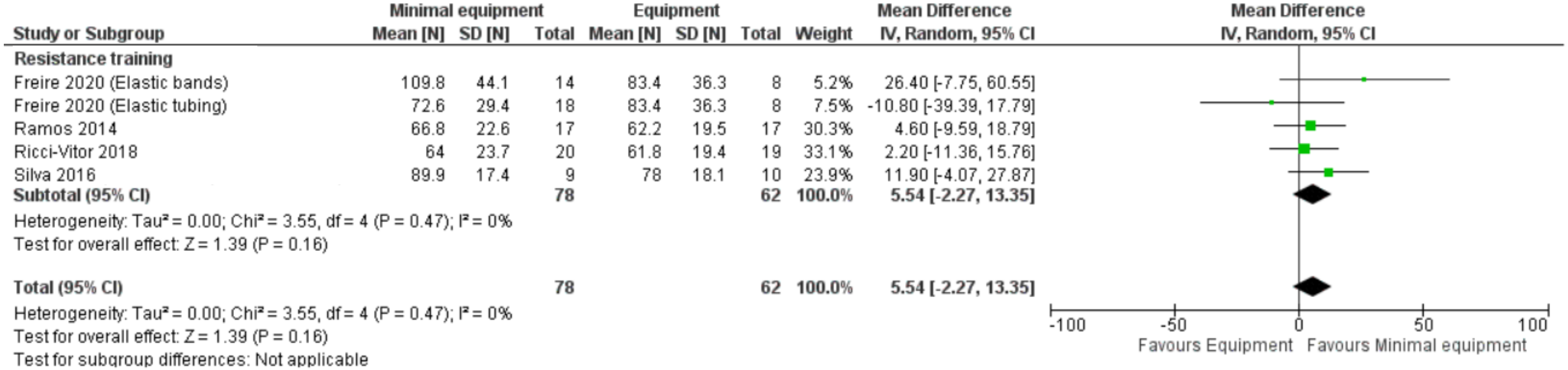

b Shoulder abduction strength

Study or Subgroup	Minimal equipment Mean [N]	SD [N]	Total	Equipment Mean [N]	SD [N]	Total	Weight	Mean Difference IV, Random, 95% CI
**Resistance training**								
Freire 2020 (Elastic bands)	115.7	50.4	14	81.4	37.3	8	4.1%	34.30 [-2.65, 71.25]
Freire 2020 (Elastic tubing)	77.5	36.3	18	81.4	37.3	8	5.9%	-3.90 [-34.71, 26.91]
Ramos 2014	59.4	20.2	17	57.7	19.5	17	31.7%	1.70 [-11.65, 15.05]
Ricci-Vitor 2018	56.9	20.4	20	56.9	19.3	19	36.3%	0.00 [-12.46, 12.46]
Silva 2016	79.5	15.8	9	72.5	19.8	10	21.9%	7.00 [-9.04, 23.04]
**Subtotal (95% CI)**			**78**			**62**	**100.0%**	**3.26 [-4.25, 10.77]**
Heterogeneity: Tau² = 0.00; Chi² = 3.44, df = 4 (P = 0.49); I² = 0%								
Test for overall effect: Z = 0.85 (P = 0.40)								
**Total (95% CI)**			**78**			**62**	**100.0%**	**3.26 [-4.25, 10.77]**
Heterogeneity: Tau² = 0.00; Chi² = 3.44, df = 4 (P = 0.49); I² = 0%								
Test for overall effect: Z = 0.85 (P = 0.40)								
Test for subgroup differences: Not applicable								

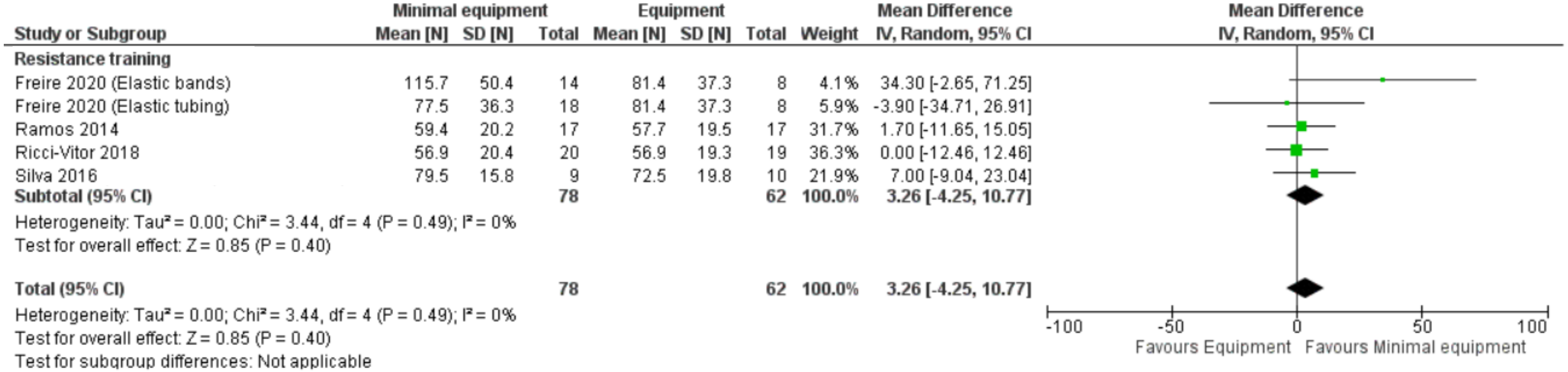

c Elbow flexion strength

	Minimal equipment			Equipment				Mean Difference
**Study or Subgroup**	**Mean [N]**	**SD [N]**	**Total**	**Mean [N]**	**SD [N]**	**Total**	**Weight**	**IV, Random, 95% CI**
**Resistance training**								
Freire 2020 (Elastic bands)	185.3	75.5	14	164.8	59.8	8	7.8%	20.50 [-36.78, 77.78]
Freire 2020 (Elastic tubing)	130.4	45.1	18	164.8	59.8	8	12.0%	-34.40 [-80.78, 11.98]
Ramos 2014	119.6	54.6	17	122.7	41.1	17	24.4%	-3.10 [-35.59, 29.39]
Ricci-Vitor 2018	107.2	55.9	20	116.8	42.8	19	26.5%	-9.60 [-40.75, 21.55]
Silva 2016	122.9	33.8	9	125.2	31.9	10	29.3%	-2.30 [-31.94, 27.34]
**Subtotal (95% CI)**			**78**			**62**	**100.0%**	**-6.48 [-22.52, 9.56]**
Heterogeneity: Tau² = 0.00; Chi² = 2.40, df = 4 (P = 0.66); I² = 0%								
Test for overall effect: Z = 0.79 (P = 0.43)								
**Total (95% CI)**			**78**			**62**	**100.0%**	**-6.48 [-22.52, 9.56]**
Heterogeneity: Tau² = 0.00; Chi² = 2.40, df = 4 (P = 0.66); I² = 0%								
Test for overall effect: Z = 0.79 (P = 0.43)								
Test for subgroup differences: Not applicable								

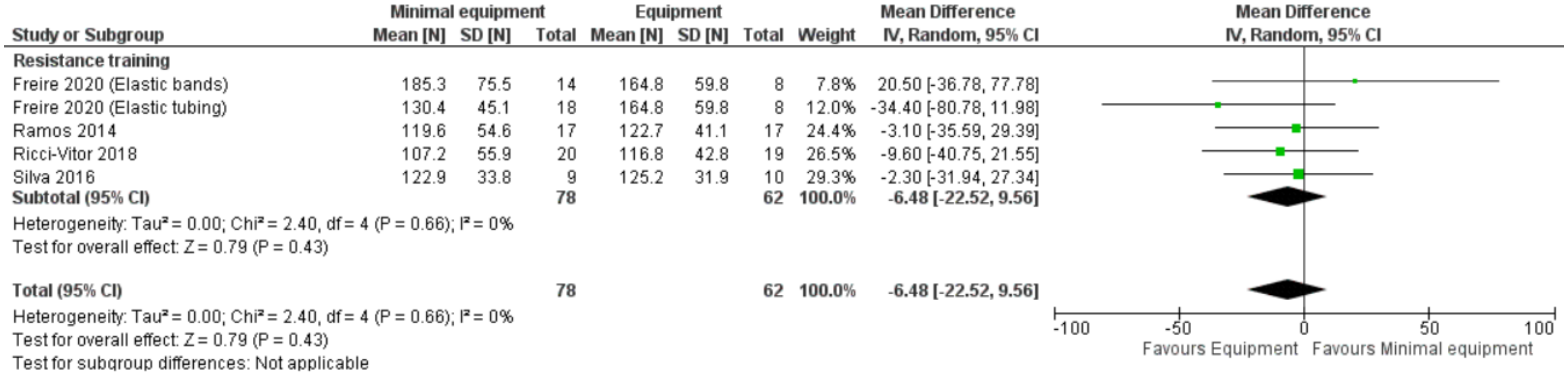

d Knee flexion strength

Study or Subgroup	Minimal equipment Mean [N]	Minimal equipment SD [N]	Minimal equipment Total	Equipment Mean [N]	Equipment SD [N]	Equipment Total	Weight	Mean Difference IV, Random, 95% CI
**Resistance training**								
Freire 2020 (Elastic bands)	319.7	105.9	14	307.9	144.2	8	11.1%	11.80 [-102.49, 126.09]
Freire 2020 (Elastic tubing)	224.6	83.4	18	307.9	144.2	8	12.0%	-83.30 [-190.39, 23.79]
Ramos 2014	130.3	34.7	17	113.4	32.3	17	27.5%	16.90 [-5.64, 39.44]
Ricci-Vitor 2018	116.8	43.3	20	115.1	30.8	19	27.3%	1.70 [-21.79, 25.19]
Silva 2016	169.5	31.2	9	288	75.1	10	22.1%	-118.50 [-169.31, -67.69]
**Subtotal (95% CI)**			**78**			**62**	**100.0%**	**-29.78 [-78.20, 18.64]**
Heterogeneity: Tau² = 2090.56; Chi² = 25.21, df = 4 (P < 0.0001); I² = 84%								
Test for overall effect: Z = 1.21 (P = 0.23)								
**Total (95% CI)**			**78**			**62**	**100.0%**	**-29.78 [-78.20, 18.64]**

Heterogeneity: Tau² = 2090.56; Chi² = 25.21, df = 4 (P < 0.0001); I² = 84%
Test for overall effect: Z = 1.21 (P = 0.23)
Test for subgroup differences: Not applicable

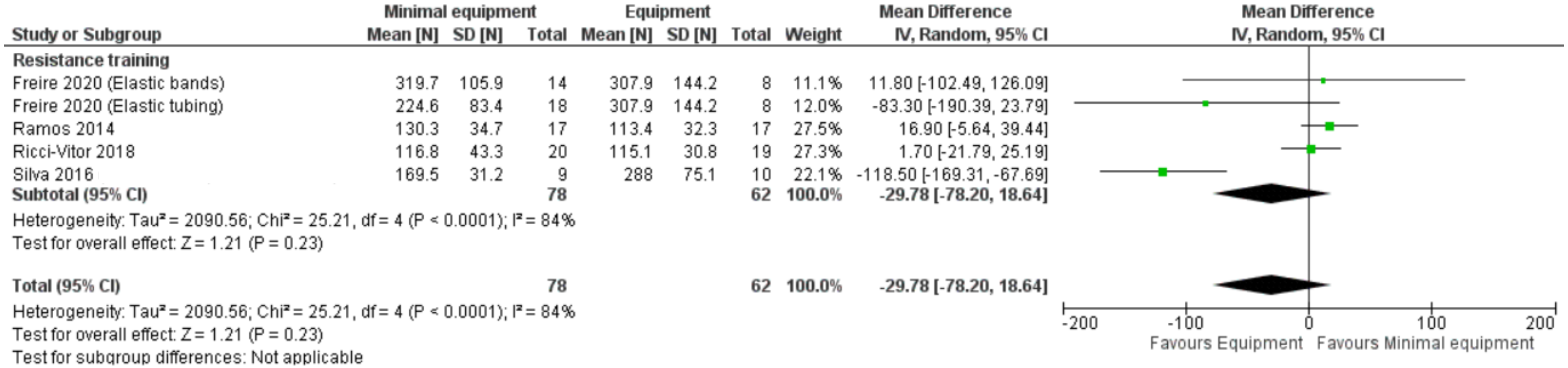

Supplement: PTJ-Data-supplement-R1mkl_pzad013 [file ptj-data-supplement-r1mkl_pzad013.pdf]
